# Platyhelminthic Cholangiopathy: A Case Report of Biliary Fascioliasis

**DOI:** 10.7759/cureus.79122

**Published:** 2025-02-16

**Authors:** Mathew Vadukoot Lazar, George S Zacharia, Mohammad Ali, Krishnamurthy Subbian, Amit Shejal, Haseena N Mohammedkunju, Hadik Patel, Divya Jose

**Affiliations:** 1 Gastroenterology, Lifecare Hospital, Abu Dhabi, ARE; 2 Gastroenterology and Hepatology, Malankara Orthodox Syrian Church Medical College, Kochi, IND; 3 Radiology, Lifecare Hospital, Abu Dhabi, ARE; 4 Pathology, Burjeel Medical City, Abu Dhabi, ARE; 5 Internal Medicine, Lifecare Hospital, Abu Dhabi, ARE; 6 Gastrointestinal Surgery, Lifecare Hospital, Abu Dhabi, ARE; 7 Nursing, Lifecare Hospital, Abu Dhabi, ARE

**Keywords:** ercp, fasciola hepatica, fascioliasis, liver flukes, triclabendazole

## Abstract

Human fascioliasis, a zoonotic infection caused by the platyhelminths *Fasciola hepatica* and *F. gigantica*, is a neglected tropical disease geographically limited to regions in South America, Africa, and Middle and South East Asia. It primarily infects mammals, particularly cattle, which serve as definitive hosts, while freshwater snails act as intermediate hosts. Human infection occurs through ingesting metacercariae found in contaminated water or aquatic plants. The disease presents with right upper abdominal pain, fever, and, at times, jaundice, hepatomegaly, and biliary obstruction. Diagnosis can be challenging due to its rarity; stool microscopy and serology often aid in diagnosis. Cross-sectional imaging and endoscopic retrograde cholangiopancreatography (ERCP) may identify the mobile helminths; the latter remains the gold standard for diagnosis. The drug of choice for fascioliasis is triclabendazole, the only drug with proven efficacy in human fascioliasis, though its availability may be limited in non-endemic regions. This case report discusses a 25-year-old male who presented with fever and right upper quadrant abdominal pain, with peripheral eosinophilia and elevated alkaline phosphatase levels. Abdominal imaging suggested biliary dilatation and sludge, while ERCP, unexpectedly multiple platyhelminths were retrieved from the biliary tree, subsequently identified as *F. hepatica*. The patient was successfully treated with two doses of oral triclabendazole, and he remained symptom-free during a six-month follow-up. This rare case highlights the diagnostic and treatment challenges of human fascioliasis outside endemic regions, offering insights into the unexpected presentation of liver flukes.

## Introduction

Human fascioliasis, a neglected tropical infection, is typically geographically localized and caused by platyhelminth parasites *Fasciola hepatica* and *F. gigantica*. It is a zoonotic infection with mammals, often cattle, forming the definitive hosts and freshwater snails, the intermediate hosts. Due to the predilection of the biliary tree, the adult worms are also referred to as liver flukes. The typical presentation is right upper abdominal pain/discomfort associated with fever. Stool microscopy and serology aid in diagnosis, the former more specific and the latter more sensitive compared to each other. Platyhelminths may be identified as mobile filling defects on abdominal imaging or during endoscopic retrograde cholangiopancreatography (ERCP). Triclabendazole is the only effective treatment and the drug of choice for treating human fascioliasis [1,2]. The limited awareness of the disease often makes it diagnostically challenging and tricky to treat. The availability of triclabendazole is also limited in non-endemic regions. The United States Food and Drug Administration (US FDA) took over 30 years to approve triclabendazole for treating fascioliasis, presumably due to the rarity or underdiagnosis of the disease in North America [3].

## Case presentation

A 25-year-old male from Ethiopia presented with right upper quadrant abdominal pain of two weeks duration. Pain was mild, constant, and non-colicky, with mild aggravation following food intake. He also reported episodes of fever with chills over a week duration. He had traveled to his home country two months prior to the presentation. He denied any yellowish discoloration of eyes, urine, or body, nor did he have any bleeding manifestations of altered mental status. Physical examination was unremarkable except for fever and mild right upper quadrant tenderness. No abdominal mass, free fluid, or palpable organomegaly was appreciated. His hemogram was normal except for eosinophilia. Serum electrolytes and renal functions were within normal limits. Hepatic biochemistry revealed elevated alkaline phosphatase (ALP) levels (Table 1).

**Table 1:** Summary of basic laboratory workup AST: aspartate aminotransferase; ALT: alanine aminotransferase; ALP: alkaline phosphatase

Parameter	Results	Reference range
Hemoglobin (gm/dL)	13.3	14-17.5
Leukocyte count (cells/μL)	6460	4400-11300
Eosinophils (%)	16.4	0-8
Platelet count (x10^3^ cells/μL)	297	150-450
Bilirubin total/direct (μmol/L)	6.1/2.5	0-21/0-5
AST/ALT (IU/L)	24/25	5-40
ALP (IU/L)	113	40-129
Lipase (IU/L)	41	13-60

**Figure 1:**
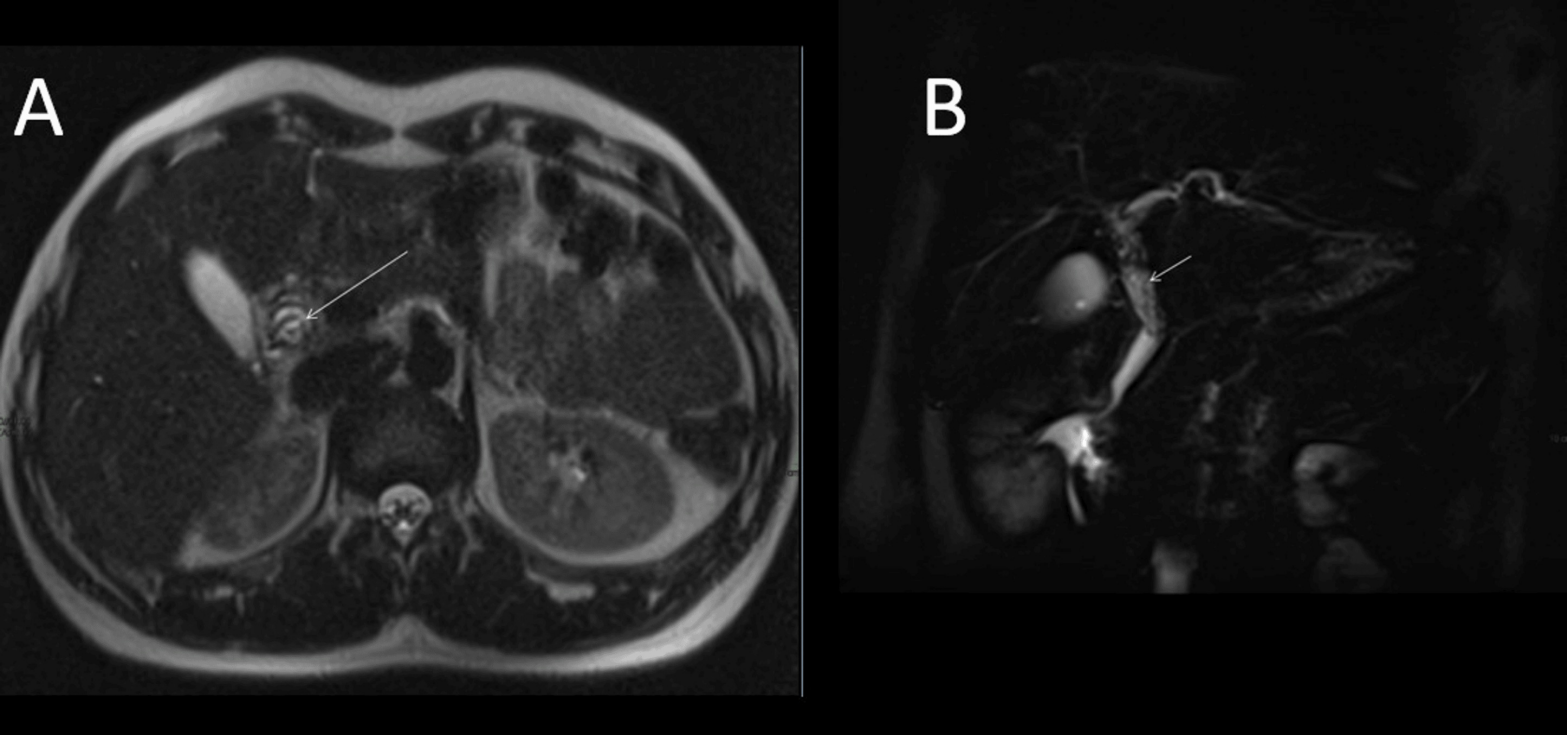
Magnetic resonance cholangiopancreatography (MRCP) images (A) Axial T2-weighted images reveal linear low-signal intensity within the common bile duct (CBD) (arrow). (B) Coronal T2 fat-saturated images also demonstrate linear low-signal intensity within the CBD (arrow).

**Figure 2:**
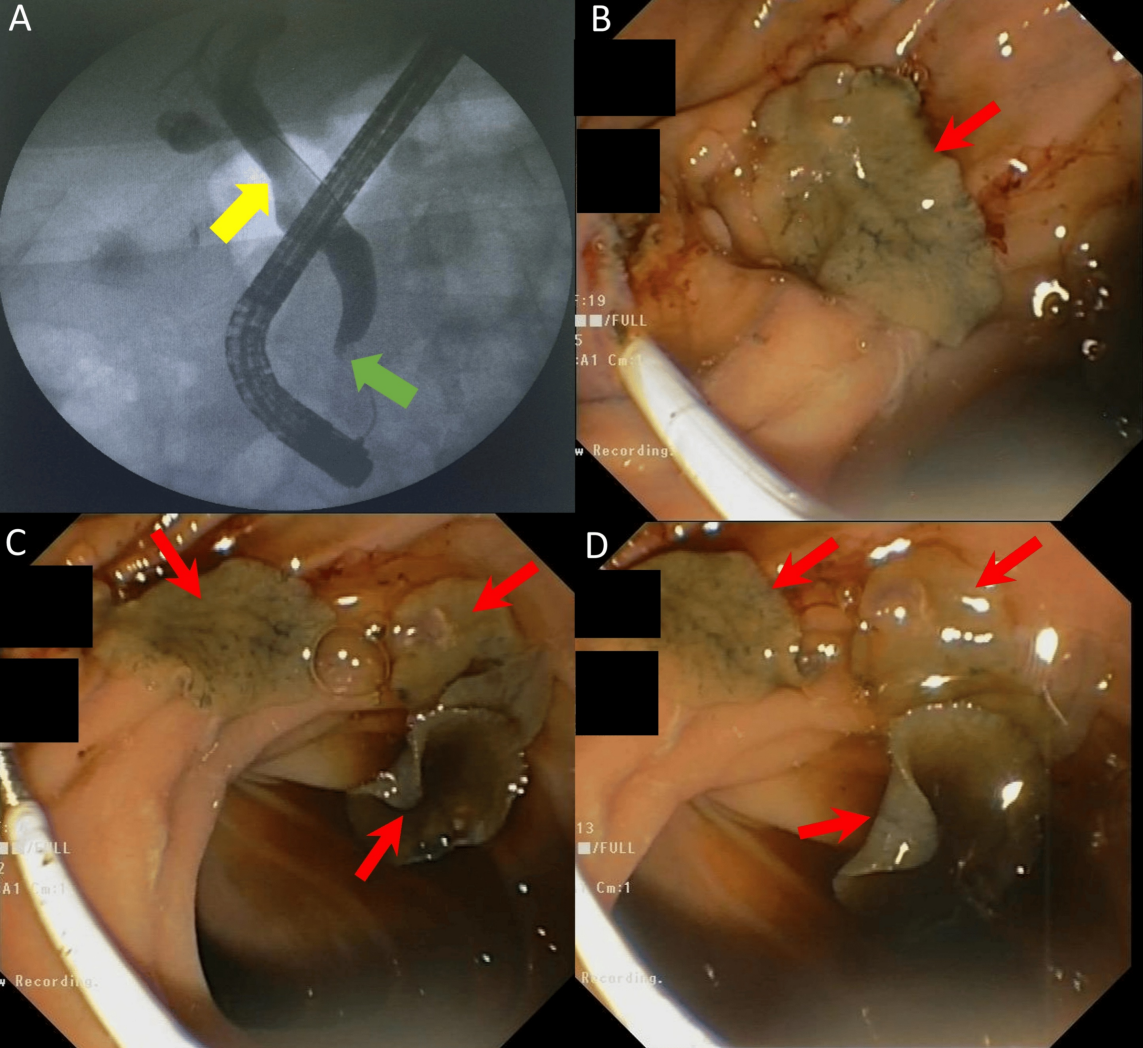
Endoscopic retrograde cholangiopancreatography (ERCP) images (A) Fluoroscopy image demonstrating a dilated common bile duct (CBD) (yellow arrow) with tapering at the distal CBD (green arrow). (B-D) Endoscopic images revealing multiple flatworms (red arrows) in the periampullary region of the duodenum.

**Figure 3:**
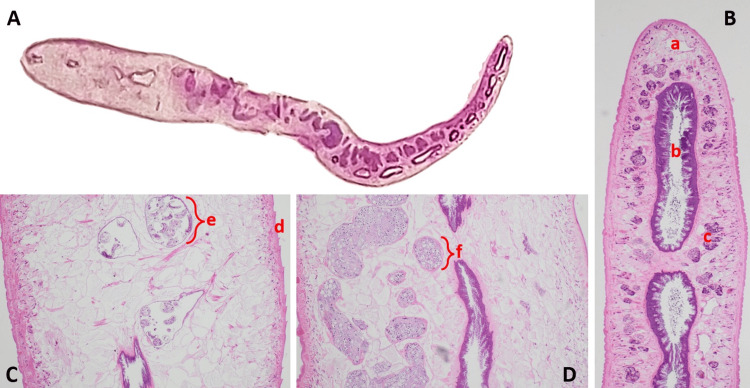
Histopathological findings (A) Fixed and sectioned whole worm of *Fasciola hepatica*. (B) Head portion (H&E stain; 400× magnification) showing a leaf-like body with an oral sucker (a), intestine (b), and vitelline follicles (c). (C) Hind portion (H&E stain; 400× magnification) demonstrating the tegument with spines (d) and a gravid uterus containing eggs (e). (D) Testis with spermatogenic cells (f).

Abdominal sonology revealed a dilated common bile duct (CBD), 9 mm (normal: <6 mm), with luminal echogenic sludge; the liver, gall bladder, and other abdominal viscera were reportedly normal. The magnetic resonance cholangiopancreatography (MRCP) demonstrated prominent common hepatic duct and CBD with intraluminal linear low-intensity signals with inflammatory thickening and gall bladder sludge (Figure 1). On ERCP, a cholangiogram confirmed the biliary dilatation, with distal CBD thickening and distal filling defect. Multiple greenish-brown leaf-shaped mobile organisms were unexpectedly swept down to the duodenal lumen during balloon extraction (Figure 2). They were retrieved with a basket for assessment, and CBD stenting was performed. Subsequently, the pathology-microbiology team identified the organism as *F. hepatica* (Figure 3).

The patient had an uneventful hospital course and was administered two doses of triclabendazole, 10mg/kg each. The biliary stent was removed after two weeks, and a balloon sweep was cleared. He remained under follow-up for almost six months with no subsequent symptoms.

## Discussion

Fascioliasis is a rare human helminthic infection included in the list of neglected tropical diseases by the World Health Organization [4]. The rare zoonotic disease is caused by flatworms or liver flukes, namely *F. hepatica* and *F. gigantica*. *F. hepatica* is widely distributed, but about half of human infections worldwide are reported from the South American countries Bolivia, Peru, and Ecuador. The Andean highlands reportedly have the world’s highest burden of fascioliasis. On the contrary, *F. gigantica* is exceptionally rare in Latin America and reported more frequently across Africa and Asia's tropical and subtropical regions [1]. Fascioliasis is a more significant concern for veterinarians as infection is more frequent among livestock [5,6]. Poverty, school-age children, consumption of uncooked vegetables and untreated water, contact with livestock, and poor sanitation have been linked with the incidence of fascioliasis [1].

The life cycle of *Fasciola* is quite complex, requiring definitive and intermediate hosts for its completion. Mammals, often cattle and sheep, are the definitive hosts, but all grazing animals and humans can hold this role. Snails belonging to the *Lymnaeidae* family, most frequently *Galba truncatula*, form the intermediate hosts. Mammals acquire the infection following ingesting the infective form, metacercariae, found free in water or attached to aquatic plants or contaminated vegetation/food. Following ingestion, they excyst in the intestine to release parasites that penetrate and migrate the intestinal wall, abdominal cavity, and hepatic parenchyma to reach their destiny in the biliary tree, where they mature into adult liver flukes. The adults release eggs, which are shed through the fecal matter that contaminates freshwater sources. In freshwater, the eggs hatch to release a miracidium, which penetrates into the freshwater snails, the intermediate hosts. In the snails, the miracidium proceeds its life cycle, sporocyst, rediae, and cercariae, each miracidium generating several cercariae. Leaving the snails, these cercariae are free-swimming until they find a suitable substrate like aquatic vegetation, then encyst to form metacercariae. Consumption of metacercariae by the definitive hosts propagates the life cycle [1-6].

Infection can be asymptomatic or symptomatic; frequent manifestations include right upper abdominal pain and fever. Others include jaundice, hepatomegaly, and, less frequently, frank cholangitis, pancreatitis, or biliary cirrhosis. Patients may develop an immune-mediated response to the helminthic antigens, resulting in rare instances of hypersensitivity pneumonitis, myocarditis, lymphadenopathy, and cerebral vasculitis, contributing to seizures or focal neurological deficits. The parasitic migration can contribute to ascites, hepatic subcapsular hematoma, hemobilia, extrahepatic inflammatory nodules, and abscess formation; however, these are rare manifestations, mostly limited to case reports, though they can confuse the clinical presentation. The initial symptoms, or the acute phase, are mainly related to the parasitic migration or acute inflammatory response, while the delayed or chronic phase is primarily due to adult worms obstructing the biliary tree or chronic inflammatory changes [2,5,7].

Diagnosis of fascioliasis can be challenging, especially in areas with only sporadic cases. Demonstration of fasciolid eggs in the stools allows definitive diagnosis. Light microscopic examination of stools may reveal the eggs; however, the sensitivity of the method is poor. Low stool egg detection rates often preclude the utility of stool analysis as a reliable test for the diagnosis of fascioliasis. Multiple stool analyses, often with concentrated specimens, may be required to enable ova identification. A combination of serology and imaging, including ERCP, typically allows for a more accurate diagnosis [8]. Serology enables the detection of anti-*Fasciola* antibodies in the blood, but the tests may remain positive after successful treatment [9]. Aspirates from duodenum may also reveal ova, but a negative result will not rule out fascioliasis, as the ova release can be intermittent [10]. Cross-sectional abdominal and ERCP allow diagnosis in most cases [5]. ERCP is considered the gold standard imaging modality for diagnosis, though not often done for diagnostic purposes, given the invasive nature and potential complications. A retrospective study by Kabaalioglu et al. reported the frequent cross-sectional imaging findings in biliary fascioliasis: helminths in the biliary tree (37%), dilated CBD (23%), intrahepatic biliary dilatation (21%), periportal adenopathy (51%), and splenomegaly (22%) [11].

The differentials to biliary fascioliasis include other parasitic infections like *Clonorchis sinensis*, *Opisthorchis viverrini*, *O. felineus*, and *Ascaris lumbricoides*. The clinical, biochemical, and radiology findings are similar across the parasites, but the gross appearance at endoscopy and microscopic features differ, allowing for correct speciation. Also, the geographic distribution varies across the parasitic species; for example, *Clonorchis* is endemic in China, Korea, Taiwan, and Vietnam; *O. viverrini* is endemic in Laos and Thailand; *O. felineus* in Eastern Europe. However, ascariasis is widely distributed, though most frequent along tropical and subtropical Asia, Africa, and the Americas. Non-parasitic causes of cholangiopathy include sclerosing cholangiopathies, ischemic and portal cavernoma cholangiopathy, bacterial cholangitis, typically secondary to calculus biliary obstruction or iatrogenic stricture, and cholangiocarcinoma [12,13].

Triclabendazole is the drug of choice in human fascioliasis. The drug has been in use for human infection since 1997 in Egypt but was approved by the US FDA for human use only in 2019. Triclabendazole is a narrow-spectrum antihelminthic agent with efficacy against *F. hepatica*, *F. gigantica*, and *Paragonimus*. The dose recommended by WHO and US FDA is 10 mg/kg, two doses, 12 to 24 hours apart. Other agents of probable benefit in human fascioliasis are nitazoxanide, metronidazole, praziquantel and artemisinin derivartives [3,5,14].

Our patient presented with clinical features compatible with *Fasciola* infection, with peripheral eosinophilia and ALP elevation. Imaging revealed biliary pathology, which was confirmed to result from fascioliasis, after the demonstration of the platyhelminth on ERCP and *F. hepatica* during the microscopic assessment. He was treated successfully with triclabendazole. Even though the diagnosis and treatment were fruitful, the findings following biliary balloon clearance were genuinely unexpected and probably a once-in-a-lifetime experience for those clinicians beyond the geographically localized high-incidence areas.

## Conclusions

Human fascioliasis is a rare zoonotic biliary infection caused by platyhelminths of the species *Fasciola*. Owing to its rarity, diagnosis can be challenging outside endemic regions. However, fascioliasis needs to be excluded in all patients with unexplained biliary pathology, even outside high-incidence areas. Triclabendazole is the drug of choice in treating fascioliasis. Improved awareness, a high index of suspicion, and targeted evaluation often allow prompt diagnosis and therapy.
